# Post-traumatic stress disorder and risk of all-cause and cause-specific mortality: a nationwide population and sibling-controlled cohort study in Taiwan

**DOI:** 10.1017/S2045796026100481

**Published:** 2026-03-03

**Authors:** Chih-Wei Hsu, Yang-Chieh Brian Chen, Liang-Jen Wang, Mu-Hong Chen, Yao-Hsu Yang, Chih-Sung Liang, Edward Chia-Cheng Lai

**Affiliations:** 1 Department of Psychiatry, Kaohsiung Chang Gung Memorial Hospital and Chang Gung University College of Medicine, Kaohsiung, Taiwan; 2 Department of Psychiatry and Behavioral Sciences, The University of Texas Health Science Center at Houston, Houston, TX, USA; 3 Department of Child and Adolescent Psychiatry, Kaohsiung Chang Gung Memorial Hospital, Chang Gung University College of Medicine, Kaohsiung, Taiwan; 4 Department of Psychiatry, Taipei Veterans General Hospital, Taipei, Taiwan; 5 Department of Psychiatry, College of Medicine, National Yang Ming Chiao Tung University, Taipei, Taiwan; 6 Department of Traditional Chinese Medicine, Chiayi Chang Gung Memorial Hospital, Chiayi, Taiwan; 7 Health Information and Epidemiology Laboratory of Chang Gung Memorial Hospital, Chiayi, Taiwan; 8 School of Traditional Chinese Medicine, College of Medicine, Chang Gung University, Taoyuan, Taiwan; 9 Department of Psychiatry, Beitou Branch, Tri-Service General Hospital, National Defense Medical University, Taipei, Taiwan; 10 Department of Psychiatry, National Defense Medical University, Taipei, Taiwan; 11 School of Pharmacy, Institute of Clinical Pharmacy and Pharmaceutical Sciences, College of Medicine, National Cheng Kung University, Tainan, Taiwan; 12 Population Health Data Center, National Cheng Kung University, Tainan, Taiwan

**Keywords:** accidents, death, mortality, PTSD, suicide

## Abstract

**Aims:**

Post-traumatic stress disorder (PTSD) may shorten life expectancy, but evidence for Asian populations and cause-specific mortality remains limited. The aim of this study is to investigate the association between PTSD and mortality risk in an Asian population.

**Methods:**

We used Taiwan’s National Health Insurance Research Database (2000–2022) to assemble a cohort of 28,777 individuals with incident PTSD and 115,108 age- and sex-matched unexposed individuals, plus a sibling cohort of 13,305 affected patients and 22,030 unaffected siblings. Cox models estimated adjusted hazard ratios (AHRs) for all-cause, unnatural-cause (suicide and accidents) and natural-cause mortality, with progressive adjustment for sociodemographic factors, comorbidity and familial confounding. Subgroup analyses addressed five psychiatric comorbidities, sex and age (youth, adulthood and older adults).

**Results:**

Over a mean follow-up of 8 years, PTSD was associated with excess all-cause mortality (AHR = 1.32, 95% CI 1.24–1.41) driven by markedly increased unnatural deaths (AHR = 5.93, 5.13–6.85), especially suicide (AHR = 10.36, 8.41–12.76) and accidental deaths (AHR = 2.18, 1.67–2.86). Natural-cause mortality showed no consistent increase (AHR = 0.91, 0.85–0.98). In sibling analyses, excess risks persisted for all-cause (AHR = 2.48, 2.04–3.01), unnatural deaths (AHR = 4.76, 3.58–6.34) and suicide mortality (AHR = 7.90, 5.21–11.97), but not for accidents or natural causes. The risk patterns were similar across different psychiatric comorbidity strata and genders; suicide and unnatural-cause excess remained evident in all age groups.

**Conclusions:**

PTSD was associated with elevated premature death risk in Taiwan, primarily through suicide and unnatural causes. Integrating targeted suicide-prevention into PTSD care pathways may be essential to reducing this avoidable mortality burden.

## Introduction

Post-traumatic stress disorder (PTSD) develops after experiencing or witnessing traumatic events and is characterized by intrusive recollections, avoidance, negative mood–cognition alterations and hyperarousal (Brewin *et al.*, 2025). It induces lasting distress and functional disability, imposing a sizeable burden on individuals, families and health systems (Brewin *et al.*, 2025). Global prevalence varies markedly: large epidemiologic surveys have shown lifetime rates of 6.9–9.2% in North American countries such as the United States and Canada, 2.2–3.9% in Western European countries such as Spain and France, but only 0.3–1.7% in Asian countries such as China, South Korea and Japan (Koenen *et al.*, 2017). These differences likely reflect variation in trauma exposure, cultural stigma and diagnostic methodology (Breslau *et al.*, 2004). Even with lower point estimates in Asia, PTSD remains a pressing public-health challenge due to its chronic course, diminished quality of life and high service utilization (Yehuda *et al.*, 2015).

A growing body of observational research links PTSD to premature mortality. Population-based studies from the United States (Schlenger *et al.*, 2015; Forehand *et al.*, 2019), Sweden (Tian *et al.*, 2022), Denmark (Gradus *et al.*, 2015) and Japan (Li *et al.*, 2019) report 10–100% excess all-cause mortality, and a recent meta-analysis indicates a 30–50% overall increase (Nilaweera *et al.*, 2023). The excess is most pronounced for suicide (one of the unnatural causes), which was almost 2–3 times higher (Forehand *et al.*, 2019; Fox *et al.*, 2021). Evidence for accidental deaths – such as poisoning, traffic injuries and falls – is limited, though a large U.S. veteran cohort found nearly a two-fold increase versus matched controls (Forehand *et al.*, 2019). Data on natural-cause mortality are few and inconsistent: some studies show higher cardiovascular, metabolic and infectious mortality (hazard ratios [HRs] 1.3–2.0) (Boscarino, 2006; Giesinger *et al.*, 2020; Kim *et al.*, 2022), whereas others, particularly those involving older adults, find no significant excess of heart disease, cancer or threatening infections (Forehand *et al.*, 2019; Song *et al.*, 2019). However, a systematic review found that these studies on causes of death related to PTSD focused mostly on male veterans in Western settings, leaving females, civilians and non-Western populations underrepresented (Nilaweera *et al.*, 2023). Moreover, nearly all studies used pre-2019 cohorts, so the potential effect of the coronavirus disease 2019 (COVID-19) pandemic on PTSD-related mortality remains unclear. Collectively, current evidence points to an elevated risk of PTSD-related mortality, but critical gaps in specific subgroups – especially Asian populations and females – underscore the need for broader and methodologically rigorous investigations.

To address these gaps, we conducted a nationwide registry-based cohort study in Taiwan. We identified every individual diagnosed with PTSD, matched each patient to population comparators on age and sex and additionally included unaffected siblings to control for shared familial factors. We quantified all-cause mortality, deaths from unnatural causes (suicide and accidents) and deaths from natural causes. We also evaluated the potential impact of COVID-19, accounted for psychiatric comorbidity and performed stratified analyses by sex and age groups.

## Methods

This population-based retrospective cohort study adhered to the REporting of studies Conducted using Observational Routinely-collected health Data (RECORD) guidelines (Appendix) (Benchimol *et al.*, 2015). Ethical approval was obtained from the Institutional Review Board of Chang Gung Memorial Hospital (CGMH IRB No. 202300262B0), with a waiver of informed consent because only de-identified secondary data were used.

### Data sources and study design

We drew on the Taiwan National Health Insurance Research Database (NHIRD), which has captured virtually every reimbursed outpatient and inpatient encounter in Taiwan (>99% of residents) since its launch in 1996 (Carvalho *et al.*, 2024; Hsu *et al.*, 2025). The NHIRD contains encrypted personal identifiers, permitting deterministic linkage across claims and the national death registry while preserving confidentiality. Available variables include sex, date of birth, residential township, income-indexed insurance premium, family relationships and diagnostic codes. Taiwan’s healthcare system used the International Classification of Diseases, Ninth Revision, Clinical Modification (ICD-9-CM) through 31 December 2015 and transitioned to the Tenth Revision, Clinical Modification (ICD-10-CM) on 1 January 2016.

**Figure 1.**
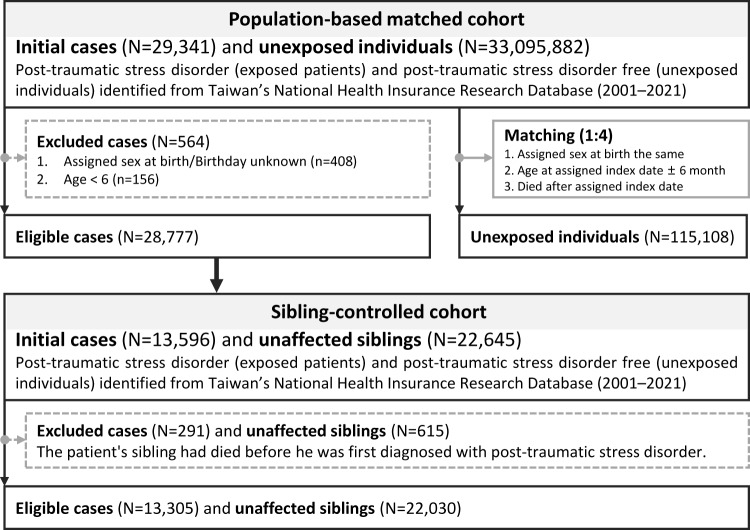
Flowchart of the selection process for the cohort study.

From 33,125,223 individuals registered between 1 January 2000 and 31 December 2022, we identified exposed patients as those who received ≥2 psychiatrist-recorded diagnoses of PTSD on separate visit dates (ICD-9-CM 309.81; ICD-10-CM F43.1) (Cheng *et al.*, 2024). As illustrated in Figure 1, 29,341 persons received an incident PTSD diagnosis between 1 January 2001 and 31 December 2021. We excluded 408 individuals with unknown sex or birthdate and 156 who were <6 years old, leaving 28,777 exposed patients for analysis. For each exposed patient, we randomly selected four population comparators without PTSD in the NHIRD records, matched on sex and date of birth (±6 months) and assigned them the same index date, yielding 115,108 unexposed individuals. To address familial confounding, we also built a sibling cohort. We identified all sibling clusters (individuals sharing ≥1 parent) in which at least one member had PTSD (n = 22,645). Siblings who had died before the proband’s first PTSD diagnosis were excluded, as they were no longer at risk during the observation period and could not serve as valid contemporaneous comparators. The final sibling cohort comprised 13,305 exposed patients and 22,030 unaffected siblings. Participants in both cohorts were followed from the index date to the earliest occurrence of death, emigration or 31 December 2022. For population comparators, the index date was the proband’s first PTSD diagnosis to ensure temporal comparability. For unaffected siblings, we used the start of NHIRD coverage for PTSD case identification (1 January 2001) as the index date due to the absence of a natural index event, providing an objective starting point while maintaining temporal consistency with our study period (Fernández de la Cruz *et al.*, 2024). This design enabled parallel estimation of population-level associations and within-family comparisons, effectively controlling for unmeasured genetic and environmental confounders shared among siblings.

### Outcomes and covariates

All-cause mortality was defined as any death recorded in the NHIRD Death Registry. Cause-specific mortality was classified according to the certified underlying cause of death using the death registry’s standardized categorizations, which distinguishes between unnatural causes (including suicide and accidental injury) and natural causes. The primary and secondary outcomes were all-cause mortality and deaths attributed to unnatural or natural causes, respectively. As suicide and accidental injury account for the majority of unnatural deaths in Taiwan, these two subcategories were also examined.

Residential urbanization and individual income served as indicators of socioeconomic position. Urbanization was classified with the Ministry of the Interior township-level index – which integrates population density, age structure, proportion of agricultural workers, tertiary-education prevalence and physician density – and was collapsed into four categories (level 1 = most urbanized; level 4 = most rural), consistent with prior NHIRD studies (Lin *et al.*, 2011; Tsai *et al.*, 2017). Monthly National Health Insurance premiums, which are directly linked to wage brackets, were converted into annual income quartiles (highest, upper-middle, lower-middle, lowest) using the distribution among matched unexposed group; this approach reliably captures socioeconomic gradients in Taiwan’s universal-coverage system (Cho *et al.*, 2019). Missing values for income or urbanization were infrequent and were retained in the analysis as an ‘unknown’ category to avoid selection bias. Somatic disease burden was summarized with the Charlson Comorbidity Index (CCI), which encompasses 22 chronic conditions and shows excellent discrimination for long-term mortality in the NHIRD (Charlson *et al.*, 1987; Quan *et al.*, 2011). Psychiatric disorders were categorized a priori into five mutually exclusive groups – psychotic disorders, bipolar disorders, depressive disorders, anxiety disorders and substance use disorders – because each cluster has been linked to elevated mortality risk (Cuijpers *et al.*, 2014; Meier *et al.*, 2016; Larney *et al.*, 2020; Correll *et al.*, 2022; Biazus *et al.*, 2023). ICD‐9-CM and ICD-10-CM codes for these psychotic disorders are listed in eTable 1. To ensure consistency in covariate ascertainment, both physical and psychiatric comorbidities were defined using diagnoses recorded within 1 year before the index date. For the sibling cohort, birth order was extracted for each individual to allow exploration of within-family gradients in mortality risk.

### Statistical analyses

Baseline characteristics of the population-matched cohort and the sibling cohort were summarized separately; continuous variables were reported as means with standard deviations (SDs), and categorical variables as absolute numbers with corresponding percentages. In the population-matched cohort, cumulative incidence was displayed using the Aalen-Johansen estimator to appropriately account for competing risks in cause-specific mortality analyses. Mortality risk was estimated using Cox proportional hazards models that employed time since cohort entry as the underlying time scale. We presented HRs with 95% confidence intervals (CIs). An initial unadjusted model provided crude HR (CHR, model 1), whereas a fully adjusted model controlled for sex, birth year, township-level urbanization, income quartile and the CCI (adjusted HR [AHR], model 2).

To evaluate the robustness of these estimates, we conducted two sensitivity analyses. A first analysis excluded participants with missing covariate data to maintain cases with complete data, while a second analysis removed person-time accrued on or after 1 January 2020 to mitigate potential bias introduced by the COVID-19 pandemic. We then repeated the main Cox models in the sibling cohort, retaining the unadjusted specification (model 1) and extending the adjusted specification to include birth order in addition to the variables listed above (model 3), thereby accounting for both measured and shared familial factors. The inclusion of birth order as a covariate was particularly important given prior evidence suggesting that birth order may independently influence mortality risk (Barclay and Kolk, 2015).

Within the matched cohort, we carried out three additional exploratory analyses. Firstly, to assess the independent association between PTSD and mortality while accounting for psychiatric comorbidities, we fitted a fully adjusted Cox model that simultaneously included all five psychiatric comorbidity categories in addition to the covariates in model 2. This model (model 4) provides HR estimates for both PTSD and each psychiatric comorbidity; for individuals with multiple conditions, the combined mortality risk can be estimated by multiplying the respective HRs. Secondly, we obtained sex-specific HRs by stratifying the data and refitting both unadjusted and adjusted models (model 1 and model 2). Thirdly, we examined age heterogeneity by estimating crude and adjusted HRs (model 1 and model 2) separately for children and adolescents (6–18 years), working-age adults (18–65 years) and older adults (≥65 years). All statistical analyses were performed with SAS software, version 9.4 (SAS Institute, Cary, North Carolina), and statistical significance was defined as a two-sided P-value less than 0.05.

## Results

In the population-based matched cohort, we identified 28,777 individuals diagnosed with PTSD and 115,108 sex- and age-matched unexposed individuals. Mean follow-up time was similar in the two groups – 8.4 years (SD 5.7) for the PTSD group and 8.5 years (SD 5.7) for the unexposed group. As detailed in Table 1, the average age at first PTSD diagnosis was 36.4 years (SD 15.7), and 75.7% of affected individuals were female. Relative to their matched counterparts, patients with PTSD were more likely to fall into the lowest income quartile (31.7% vs 22.3%), to carry a heavier somatic disease burden (CCI ≥ 1, 30.4% vs 17.2%) and to present with comorbid psychiatric disorders (psychotic disorders, 2.3% vs 0.5%; bipolar disorders, 3.4% vs 0.3%; depressive disorders, 26.9% vs 1.3%; anxiety disorders, 15.3% vs 1.1%; and substance-use disorders, 1.0% vs 0.1%). A similar pattern emerged in the sibling cohort, comprising 13,305 affected individuals and 22,030 unaffected siblings, although between-group differences were attenuated for somatic disease burden and comorbid psychiatric disorders.

**Table 1.** Characteristics of all included participants from 2001 to 2021

	Population-based matched cohort		Population-based sibling cohort	
Characteristics	PTSD (n = 28,777)	Unexposed group (n = 115,108)	PTSD (n = 13,305)	Unaffected siblings (n = 22,030)
**Basic information**				
Sex, female	21,787 (75.7)	87,148 (75.7)	9731 (73.1)	10,719 (48.7)
Age, year	36.4 ± 15.7	36.4 ± 15.7	–	–
**Personal urbanization level**				
Level 1 (most urbanized)	15,853 (55.1)	64,123 (55.7)	7380 (55.5)	11,852 (53.8)
Level 2	10,715 (37.2)	40,461 (35.2)	4964 (37.3)	8389 (38.1)
Level 3	1625 (5.6)	7544 (6.6)	725 (5.4)	1369 (6.2)
Level 4 (most rural)	316 (1.1)	1058 (0.9)	135 (1.0)	217 (1.0)
Unknown	268 (0.9)	1922 (1.7)	101 (0.8)	203 (0.9)
**Personal income level**				
<25% (highest)	5060 (17.6)	26,368 (22.9)	2136 (16.1)	4505 (20.4)
25–50% (upper-middle)	6317 (22.0)	27,034 (23.5)	3434 (25.8)	5364 (24.3)
50–75% (lower-middle)	6637 (23.1)	26,172 (22.7)	2759 (20.7)	4293 (19.5)
≥75% (lowest)	9119 (31.7)	25,686 (22.3)	4106 (30.9)	5670 (25.7)
Unknown	1644 (5.7)	9848 (8.6)	870 (6.5)	2198 (10.0)
**Somatic diseases**				
0 (Charlson Comorbidity Index)	20,023 (69.6)	95,289 (82.8)	10,687 (80.3)	19,484 (88.4)
1–2	7410 (25.7)	16,848 (14.6)	2426 (18.2)	2386 (10.8)
≥3	1344 (4.7)	2971 (2.6)	192 (1.4)	160 (0.7)
**Psychiatric disorders**				
Psychotic disorders	666 (2.3)	537 (0.5)	332 (2.5)	107 (0.5)
Bipolar disorders	968 (3.4)	290 (0.3)	408 (3.1)	84 (0.4)
Depressive disorders	7745 (26.9)	1537 (1.3)	3168 (23.8)	402 (1.8)
Anxiety disorders	4392 (15.3)	1212 (1.1)	1822 (13.7)	320 (1.5)
Substance use disorders	289 (1.0)	80 (0.1)	101 (0.8)	32 (0.1)

Abbreviation: PTSD, post-traumatic stress disorder.

Data were expressed as mean (standard deviation) or N (percentage).

During follow-up, 1414 deaths (4.9%) occurred in the PTSD group and 3485 deaths (3.0%) in the unexposed group, corresponding to crude mortality rates of 19.6 and 12.2 per 10,000 person-years, respectively (Table 2). Cumulative incidence curves (eFigure 1A–E) illustrate consistently higher mortality in the PTSD group. In Cox models, PTSD was associated with excess all-cause mortality both before and after covariate adjustment (CHR 1.65, 95% CI 1.56–1.76; AHR 1.30, 95% CI 1.22–1.39). The elevation was more pronounced for deaths from unnatural causes (CHR 6.26, 95% CI 5.45–7.19; AHR 5.47, 95% CI 4.74–6.32), especially suicide (CHR 10.71, 95% CI 8.76–13.09; AHR 9.88, 95% CI 8.04–12.14). Accidental mortality was also higher in the PTSD group (CHR 2.36, 95% CI 1.82–3.06; AHR 1.94, 95% CI 1.48–2.54). By contrast, the association with natural-cause mortality was inconsistent: the crude model suggested modest excess risk (CHR 1.17, 95% CI 1.09–1.26), whereas the adjusted model indicated a slightly reduced risk (AHR 0.89, 95% CI 0.83–0.96). Two sensitivity analyses limited to complete cases and to person-time accrued before 2020 produced virtually identical results.

**Table 2.** Risk of all-cause and cause-specific mortality in post-traumatic stress disorder group and unexposed group

Characteristics	PTSD,event	PTSD,mortality rate	Unexposed group,event	Unexposed group,mortality rate	Crude hazard ratio (model 1)	Adjusted hazard ratio (model 2)^1^
**Primary analysis**	(n = 28,777)		(n = 115,108)			
All-cause	1414 (4.9)	58.6	3485 (3.0)	35.6	1.65 (1.56–1.76)*	1.30 (1.22–1.39)*
Unnatural causes	506 (1.8)	21.0	328 (0.3)	3.3	6.26 (5.45–7.19)*	5.47 (4.74–6.32)*
Suicides	346 (1.2)	14.3	131 (0.1)	1.3	10.71 (8.76–13.09)*	9.88 (8.04–12.14)*
Accidents	90 (0.3)	3.7	155 (0.1)	1.6	2.36 (1.82–3.06)*	1.94 (1.48–2.54)*
Natural causes	908 (3.2)	37.6	3157 (2.7)	32.2	1.17 (1.09–1.26)*	0.89 (0.83–0.96)*
**Sensitivity analysis(excluding missing data)**	(n = 26,902)		(n = 104,231)			
All-cause	1292 (4.8)	56.2	3004 (2.9)	33.0	1.71 (1.60–1.83)*	1.33 (1.24–1.42)*
Unnatural causes	481 (1.8)	20.9	302 (0.3)	3.3	6.30 (5.46–7.28)*	5.48 (4.73–6.35)*
Suicides	328 (1.2)	14.3	121 (0.1)	1.3	10.72 (8.70–13.20)*	9.75 (7.88–12.07)*
Accidents	84 (0.3)	3.7	142 (0.1)	1.6	2.35 (1.79–3.07)*	1.93 (1.46–2.55)*
Natural causes	811 (3)	35.3	2702 (2.6)	29.7	1.20 (1.11–1.29)*	0.89 (0.82–0.97)*
**Sensitivity analysis(excluding data after 2020)**	(n = 20,891)		(n = 83,564)			
All-cause	950 (4.5)	58.5	2309 (2.8)	35.0	1.68 (1.55–1.81)*	1.30 (1.20–1.41)*
Unnatural causes	354 (1.7)	21.8	226 (0.3)	3.4	6.35 (5.37–7.50)*	5.51 (4.63–6.54)*
Suicides	238 (1.1)	14.7	86 (0.1)	1.3	11.21 (8.76–14.34)*	10.31 (8.01–13.28)*
Accidents	62 (0.3)	3.8	108 (0.1)	1.6	2.33 (1.71–3.18)*	1.90 (1.38–2.63)*
Natural causes	596 (2.9)	36.7	2083 (2.5)	31.6	1.17 (1.06–1.28)*	0.88 (0.80–0.96)*

Abbreviation: PTSD, post-traumatic stress disorder.

Event was expressed as N (percentage) and mortality rate was expressed as event per 10,000 person-years.

1 Model 2 was adjusted for all variables (birth year, sex, income level, urbanization level, and Charlson Comorbidity Index).

Within the sibling cohort, 260 deaths (2.0%) occurred among individuals with PTSD and 190 deaths (0.9%) among their unaffected siblings, yielding crude mortality rates of 8.9 and 10.1 per 10,000 person-years, respectively (Table 3). PTSD remained associated with elevated all-cause mortality (CHR 2.28, 95% CI 1.89–2.75; AHR 2.16, 95% CI 1.77–2.63), deaths from unnatural causes (CHR 4.48, 95% CI 3.39–5.92; AHR 4.38, 95% CI 3.27–5.86) and suicide (CHR 7.97, 95% CI 5.30–11.97; AHR 7.60, 95% CI 4.97–11.61). In contrast to the population comparison, accidental mortality did not differ significantly between patients and siblings (CHR 1.14, 95% CI 0.67–1.97; AHR 1.30, 95% CI 0.73–2.30), and no association was observed for natural-cause deaths (CHR 1.05, 95% CI 0.79–1.40; AHR 0.95, 95% CI 0.70–1.28).

**Table 3.** Risk of all-cause and cause-specific mortality in post-traumatic stress disorder group and unaffected siblings

Characteristics	PTSD,event	PTSD,mortality rate	Unaffected siblings,event	Unaffected siblings,mortality rate	Crude hazard ratio (model 1)	Adjusted hazard ratio (model 3)^1^
**Sibling cohort**	(n = 13,305)		(n = 22,030)			
All-cause	260 (2.0)	8.9	190 (0.9)	3.9	2.28 (1.89–2.75)*	2.16 (1.77–2.63)*
Unnatural causes	183 (1.4)	6.3	68 (0.3)	1.4	4.48 (3.39–5.92)*	4.38 (3.27–5.86)*
Suicides	134 (1.0)	4.6	28 (0.1)	0.6	7.97 (5.30–11.97)*	7.60 (4.97–11.61)*
Accidents	22 (0.2)	0.8	32 (0.1)	0.7	1.14 (0.67**–**1.97)	1.30 (0.73**–**2.30)
Natural causes	77 (0.6)	2.6	122 (0.6)	2.5	1.05 (0.79**–**1.40)	0.95 (0.70**–**1.28)

Abbreviation: PTSD, post-traumatic stress disorder.

Event was expressed as N (percentage) and mortality rate was expressed as event per 10,000 person-years.

1 Model 3 was adjusted for all variables (birth year, sex, income level, urbanization level, Charlson Comorbidity Index, and sibling birth order).

When we repeated the analyses within the population-matched cohort by simultaneously adjusting for all five psychiatric comorbidity categories (model 4), PTSD remained independently associated with elevated all-cause mortality (AHR 1.11, 95% CI 1.03–1.20), unnatural deaths (AHR 3.51, 95% CI 2.97–4.15), suicide (AHR 5.71, 95% CI 4.51–7.23) and accidental deaths (AHR 1.54, 95% CI 1.12–2.10) (Table 4). In contrast, PTSD was associated with reduced natural-cause mortality after full adjustment (AHR 0.84, 95% CI 0.77–0.92). These patterns were broadly consistent with the primary analysis (model 2, Table 2). Among the psychiatric comorbidities examined, depressive disorders showed the strongest associations with unnatural deaths (AHR 2.73, 95% CI 2.28–3.26) and suicide (AHR 3.48, 95% CI 2.79–4.34), while substance use disorders were associated with accidental mortality (AHR 7.10, 95% CI 3.63–13.86). Sex-stratified analyses revealed similar risk patterns in female and male (eTable 2). Age-stratified models showed that the heightened risks for deaths from unnatural causes and for suicide persisted across children and adolescents (6–18 years), adults (18–65 years) and older adults (≥65 years), whereas other mortality outcomes varied by life stage (eTable 3). Specifically, PTSD was not associated with accidental deaths in children and adolescents or older adults; PTSD showed a positive association with natural-cause mortality in children and adolescents but not in adults; and in older adults, PTSD was associated with significantly lower all-cause and natural-cause mortality.

**Table 4.** Independent risk of all-cause and cause-specific mortality associated with post-traumatic stress disorder and psychiatric comorbidities in the population-based matched cohort

Characteristics	PTSD	Psychotic disorders	Bipolar disorders	Depressive disorders	Anxiety disorders	SUD
**All-cause**	1.11 (1.03–1.20)*	1.80 (1.46–2.23)*	2.12 (1.73–2.61)*	1.39 (1.26–1.53)*	0.96 (0.84**–**1.09)	2.22 (1.66–2.97)*
**Unnatural causes**	3.51 (2.97–4.15)*	1.67 (1.19–2.35)*	2.02 (1.49–2.73)*	2.73 (2.28–3.26)*	0.99 (0.80**–**1.23)	2.00 (1.30–3.07)*
Suicides	5.71 (4.51–7.23)*	1.57 (1.03–2.39)*	2.23 (1.56–3.18)*	3.48 (2.79–4.34)*	1.00 (0.77**–**1.30)	1.06 (0.54**–**2.08)
Accidents	1.54 (1.12–2.10)*	2.04 (0.99**–**4.20)	1.72 (0.81**–**3.67)	1.49 (0.98**–**2.27)	0.86 (0.50**–**1.48)	7.10 (3.63–13.86)*
**Natural causes**	0.84 (0.77–0.92)*	1.71 (1.30–2.25)*	1.91 (1.44–2.53)*	1.08 (0.95**–**1.22)	0.97 (0.83**–**1.14)	2.24 (1.51–3.32)*

Abbreviation: PTSD, post-traumatic stress disorder; SUD, substance use disorders.

Data were expressed as adjusted hazard ratios with 95% confidence intervals.

Adjusted for all variables (birth year, sex, income level, urbanization level, Charlson Comorbidity Index and all psychiatric comorbidities).

## Discussion

In this Taiwanese population-based cohort study using a simultaneous sibling comparison design, we demonstrated that PTSD was associated with a significantly higher risk of all-cause mortality as well as mortality from unnatural causes, particularly suicide. These excess risks remained unchanged after adjustment for demographic factors and physical comorbidities (all-cause: 1.3-fold; unnatural causes: 5.5-fold; and suicide: 9.9-fold), and were robust to sensitivity analyses addressing missing data, the COVID-19 period, shared familial confounding, different sexes and psychiatric comorbidities. Compared with matched unexposed individuals, participants with PTSD also exhibited elevated mortality from accidents; however, this association was no longer observed in the sibling-controlled analyses. By contrast, the risk of deaths due to natural causes showed no consistent pattern across analytic scenarios.

When we examined the three hierarchically related outcomes – suicide, unnatural deaths and all-cause mortality – our findings echoed the previous literature: compared with the general population, people with PTSD experienced markedly higher risks at every tier (Gradus *et al.*, 2015; Schlenger *et al.*, 2015; Forehand *et al.*, 2019; Li *et al.*, 2019; Fox *et al.*, 2021; Tian *et al.*, 2022). Notably, the mortality patterns observed in Taiwan (Asian populations) – including a 30% excess in all-cause mortality and a 10-fold elevation in suicide – closely parallel estimates from these Nordic and U.S. cohorts (Forehand *et al.*, 2019; Tian *et al.*, 2022; Nilaweera *et al.*, 2023), suggesting that the PTSD–mortality association is consistent across diverse populations despite differences in trauma exposure, healthcare systems and cultural contexts. Only a handful of earlier studies have tried to isolate genetic and early-environmental influences by pairing affected individuals with their own unaffected siblings, and those few reports showed that the excess hazards persisted (Tian *et al.*, 2022). Our sibling-controlled analyses reinforced this evidence: even after accounting for shared familial factors, PTSD remained strongly associated with elevated risks of suicide, other unnatural deaths and all-cause mortality. No studies have investigated whether COVID-19 affects mortality risk in people with PTSD. We found similar results in effect estimates when follow-up during the COVID-19 period was retained (primary analysis) or excluded (sensitivity analysis removing data from 2020 onward) (Table 2), indicating that the pandemic did not meaningfully influence the results. Prior work has suggested that comorbid depression might explain much of PTSD-related excess mortality. Accordingly, we fitted a fully adjusted model that simultaneously controlled for all five psychiatric comorbidity categories (Table 4). After this adjustment, PTSD remained independently associated with elevated risks of all-cause mortality, unnatural deaths and suicide, though effect sizes were attenuated compared with the primary analysis. This pattern suggests that while psychiatric comorbidities contribute to the excess mortality observed in those with PTSD, they do not fully account for it, and PTSD itself demonstrates an independent and substantial association with premature death.

With respect to accidental mortality, our data indicate that individuals with PTSD faced more than double the risk of dying from accidents compared with their population‐matched unexposed individuals (AHR = 1.94). However, when the comparison was restricted to unaffected siblings, the excess risk decreased to roughly 1.3‐fold and was no longer statistically significant. This attenuation suggests that genetic or shared household factors – rather than PTSD alone – may explain part of the association with accidental death. One possible interpretation is that the elevated accident risk observed in population-based studies could partly reflect unmeasured familial factors, such as inherited impulsivity or risk-taking propensities, that might predispose to both PTSD and accidental injury (Stein *et al.*, 2002; Sharma and Ressler, 2019). Although previous work has attributed higher accident rates in PTSD to downstream risk behaviours such as substance or medication misuse (Boscarino, 2006; Forehand *et al.*, 2019), our findings suggest that some of these behaviours may be associated with shared family environments that foster hazardous coping responses to stress. This interpretation implies that in families without such predispositions, a PTSD diagnosis may not be associated with comparable increases in accident risk.

For natural cause mortality, PTSD showed a significant elevation in deaths from medical causes in the crude analysis, mirroring earlier studies that linked the disorder to heightened risks of cardiovascular disease, infections and other somatic conditions (Boscarino, 2006; Giesinger *et al.*, 2020; Kim *et al.*, 2022) – mechanisms often ascribed to chronic dysregulation of the hypothalamic–pituitary–adrenal axis (Yehuda *et al.*, 1996). However, after comprehensive adjustment, particularly for the CCI, the association not only diminished but reversed, with PTSD cases showing slightly lower natural-cause mortality than comparators. Several interpretations may explain this unexpected pattern. Firstly, although PTSD may be associated with aggravated cardiometabolic and infectious pathways, those effects are overshadowed when patients are compared with unexposed individuals who possess a similar burden of chronic physical illness; in other words, the excess risk of natural-cause mortality associated with PTSD may be largely absorbed by pre-existing somatic comorbidities captured in the CCI. Secondly, individuals diagnosed with PTSD are, by definition, already engaged with the healthcare system, which may facilitate earlier detection and more intensive management of emerging physical health conditions. This increased medical surveillance and treatment access could be associated with reduced mortality from natural causes (Baker *et al.*, 2020), particularly when compared with matched unexposed individuals who share a similar level of somatic disease burden but may have less frequent clinical contact.

Earlier meta-analyses have highlighted that most cohorts have been heavily male, leaving the mortality experience of females with PTSD largely unexplored (Nilaweera *et al.*, 2023). Our cohort, in which females comprised 76% of participants, reflects the higher prevalence of PTSD among women (Tolin and Foa, 2006), directly addressing this gap. Sex-stratified analyses demonstrated that the excess mortality burden is not limited to the male veteran populations that dominated prior research (Nilaweera *et al.*, 2023). Females with PTSD showed comparable or higher HRs for suicide (AHR 11.85) compared with males (AHR 7.38), underscoring that clinical vigilance for suicide risk should not be limited to male patients (eTable 2). The age-stratified findings add another layer of nuance. Across all three life-stage categories – children and adolescents, adults, and older adults – the associations with suicide and the broader category of unnatural deaths remained robust, underscoring the pervasive lethality in PTSD regardless of age. For other causes of death, however, the pattern diverged by life stage (eTable 3). Natural-cause mortality showed a positive association with PTSD in children and adolescents, no significant association in adults and a significant inverse association in older adults. Similarly, all-cause mortality showed the strongest positive association in children and adolescents but a significant inverse association in older adults. One plausible explanation for the attenuated or reversed associations in older adults is that PTSD exerts many of its downstream effects through lifelong biological and behavioural pathways – such as chronic inflammation (Sumner *et al.*, 2020; Katrinli *et al.*, 2022), disrupted neuropsychological development (Kennedy *et al.*, 2018) and gene–environment interactions (Mehta *et al.*, 2013; Ressler *et al.*, 2022) – that are initiated soon after trauma exposure. Individuals who first develop PTSD late in life may have had insufficient time for these cumulative processes to manifest, and may additionally benefit from increased healthcare engagement associated with their PTSD diagnosis.

The pronounced suicide risk observed in our study – nearly 10-fold higher than the general population and persisting after adjustment for psychiatric comorbidities – has specific clinical implications. Firstly, given the substantial contribution of depressive disorders to suicide mortality (Table 4), aggressive treatment of comorbid depression should be prioritized. Secondly, evidence-based psychotherapies such as cognitive processing therapy and prolonged exposure that directly target PTSD symptoms have been shown to reduce suicidal ideation (Gradus *et al.*, 2013), and achieving symptomatic remission may substantially lower suicide mortality (Forehand *et al.*, 2022). Thirdly, the persistence of excess suicide risk in sibling-controlled analyses suggests that family-based interventions, such as educating relatives about warning signs and fostering supportive home environments, may complement individual-level treatment.

A major strength of this study is its population-based matched cohort coupled with a simultaneous sibling-comparison design to examine PTSD-related mortality in an Asian setting. However, there are several limitations worth considering. Firstly, because the database lacks information on lifestyle factors such as smoking, diet, alcohol use and occupational exposures, we were unable to adjust for these potential non-disease determinants of mortality. Secondly, death certificates did not differentiate natural deaths into specific medical categories (e.g., cardiovascular, infectious or metabolic), constraining detailed cause-specific analyses. This limits direct comparison with prior studies that reported elevated risks for specific conditions such as cardiovascular disease or infections. While we can identify overall patterns of natural-cause mortality, we cannot replicate the granular disease-specific analyses in previous investigations. Thirdly, several sources of potential bias warrant consideration. Regarding survivor bias, our design appropriately initiates follow-up at the first PTSD diagnosis, thereby excluding pre-diagnosis deaths from attribution to PTSD. Since only post-diagnosis deaths are captured as outcomes, concerns about requiring survival until diagnosis to enter the cohort are minimized. Concerning misclassification bias, a small number of individuals with PTSD may remain undiagnosed and be misclassified into the unexposed group. However, such non-differential misclassification would bias our results toward the null, meaning that the substantial risk elevations we observed, particularly for suicide and unnatural deaths, likely represent conservative estimates. Fourthly, socioeconomic factors (urbanization and income) were controlled through statistical adjustment rather than matching. While urbanization was well-balanced between groups, patients with PTSD were overrepresented in the lowest income quartile (31.7% vs 22.3%). Matching on these variables would have reduced the sample size and potentially introduced selection bias through the exclusion of unmatchable cases. Although multivariable adjustment addressed these imbalances, residual confounding from the income difference cannot be excluded. Finally, the work was conducted within Taiwan’s single-payer healthcare system; although the findings may be informative for other Asian populations, their applicability to regions with substantially different medical infrastructures or cultural contexts remains uncertain. Reassuringly, our estimates for all-cause, unnatural-cause and suicide mortality closely parallel those reported in Nordic and U.S. cohorts, suggesting that the mortality profile associated with PTSD in Asian populations resembles that observed in many Western countries.

## Conclusion

This population-based cohort of 28,777 Taiwanese residents with PTSD showed a 30% excess in all-cause mortality versus matched unexposed individuals, an excess almost entirely accounted for by an over 5-fold elevation in deaths from unnatural causes – principally a 10-fold elevation in suicide and nearly 2-fold elevation in accidents. Sibling comparisons and psychiatric-comorbidity-adjusted models partially attenuated the accident signal and modestly narrowed the overall gap, implicating shared familial factors and co-occurring psychiatric disorders may partly explain these associations. The pattern of elevated risk for suicide and unnatural causes was similar among females and males and across age groups. Accordingly, policy and clinical practice may prioritize suicide risk-reduction strategies to curb preventable deaths and improve long-term survival for people living with PTSD.

## Supporting information

10.1017/S2045796026100481.sm001 Hsu et al. supplementary material 1 Hsu et al. supplementary material

10.1017/S2045796026100481.sm002 Hsu et al. supplementary material 2 Hsu et al. supplementary material

## Data Availability

The data of the current study would be available to the corresponding author upon reasonable request and approval from the Institutional Review Board and permission from the Taiwan Ministry of Health and Welfare.

## Supplementary material

The supplementary material for this article can be found at https://doi.org/10.1017/S2045796026100481.
