# Supplementary material for: Post-traumatic stress disorder and risk of all-cause and cause-specific mortality: a nationwide population and sibling-controlled cohort study in Taiwan
Source: Epidemiol Psychiatr Sci. 2026 Mar 3;35:e12. doi: 10.1017/S2045796026100481 (PMC12964077; doi:10.1017/S2045796026100481)
Supplement: Hsu et al. supplementary material 2 — Hsu et al. supplementary material [file S2045796026100481sup002.docx]

**Supplementary material**

**Post-traumatic Stress Disorder and Risk of All-Cause and Cause-Specific Mortality: A Nationwide Population and** **Sibling-Controlled Cohort Study in Taiwan**

**Short Title**: Mortality in PTSD

Chih-Wei Hsu, MD^a^, Yang-Chieh Brian Chen, MD^a,b*^, Liang-Jen Wang, MD^c^, Mu-Hong Chen, MD^d,e^, Yao-Hsu Yang, MD^f,g,h^, Chih-Sung Liang, MD^i,j*^, Edward Chia-Cheng Lai, PhD^k,l^

^a^ Department of Psychiatry, Kaohsiung Chang Gung Memorial Hospital and Chang Gung University College of Medicine, Kaohsiung, Taiwan

^b^ Department of Psychiatry and Behavioral Sciences, The University of Texas Health Science Center at Houston, Houston, TX, USA

^c^ Department of Child and Adolescent Psychiatry, Kaohsiung Chang Gung Memorial Hospital, Chang Gung University College of Medicine, Kaohsiung, Taiwan

^d^ Department of Psychiatry, Taipei Veterans General Hospital, Taipei, Taiwan

^e^ Department of Psychiatry, College of Medicine, National Yang Ming Chiao Tung University, Taipei, Taiwan

^f^ Department of Traditional Chinese Medicine, Chiayi Chang Gung Memorial Hospital, Chiayi, Taiwan

^g^ Health Information and Epidemiology Laboratory of Chang Gung Memorial Hospital, Chiayi, Taiwan

^h^ School of Traditional Chinese Medicine, College of Medicine, Chang Gung University, Taoyuan, Taiwan

^i^ Department of Psychiatry, Beitou branch, Tri-Service General Hospital, National Defense Medical University, Taipei, Taiwan

^j^ Department of Psychiatry, National Defense Medical University, Taipei, Taiwan

^k^ School of Pharmacy, Institute of Clinical Pharmacy and Pharmaceutical Sciences, College of Medicine, National Cheng Kung University, Tainan, Taiwan

^l^ Population Health Data Center, National Cheng Kung University, Tainan, Taiwan

^*^ Contributed equally as corresponding authors

| **Content** | **Page** |
| --- | --- |
| **eTable 1.** The diagnostic codes of exposures and all comorbidities | 1 |
| **eTable 2.** The risk of all-cause and cause-specific mortality between post-traumatic stress disorder group and unexposed group, by sex | 2 |
| **eTable 3.** The risk of all-cause and cause-specific mortality between post-traumatic stress disorder group and unexposed group, by age | 3 |
| **eFigure 1.** Cumulative incidence curves in post-traumatic stress disorder group and unexposed group | 4-8 |

**eTable 1.** The diagnostic codes of exposures and all comorbidities

| Medical comorbidities | International Classification of Diseases 9^th^ | International Classification of Diseases 10^th^ |
| --- | --- | --- |
| Post-traumatic stress disorder | 309.81 | F43.1 |
| Psychotic disorders | 295, 297, 298.1, 298.3, 298.4, 298.8, 298.9 | F20, F22-F25, F28, F29 |
| Bipolar disorders | 296.0, 296.1, 296.4–296.7, 296.80, 296.81, 296.89, 301.13 | F30, F31, F34.0 |
| Depressive disorders | 296.2, 296.3, 300.4, 311, 625.4 | F32, F33, F34.1 |
| Anxiety disorders | 300.0, 300.2, 309.21 | F40, F41, F93.0 |
| Substance use disorders | 303, 304, 305.0, 305.2, 305.3, 305.4, 305.5, 305.6, 305.7, 305.8, 305.9 | F10-F16, F18, F19 |

**eTable 2.** The risk of all-cause and cause-specific mortality between post-traumatic stress disorder group and unexposed group, by sex

| Characteristics | PTSD, event | PTSD, mortality rate | Unexposed group, event | Unexposed group, mortality rate | Crude hazard ratio (model 1) | Adjusted hazard ratio (model 2) |
| --- | --- | --- | --- | --- | --- | --- |
| Female | (n = 21,787) |  | (n = 87,148) |  |  |  |
| All-cause | 918 (4.2) | 49.7 | 2114 (2.4) | 28.2 | **1.77 (1.64****–1.91)*** | **1.42 (1.31–1.54)*** |
| Unnatural causes | 363 (1.7) | 19.6 | 205 (0.2) | 2.7 | **7.18 (6.05–8.52)*** | **6.20 (5.20–7.40)*** |
| Suicides | 263 (1.2) | 14.2 | 87 (0.1) | 1.2 | **12.24 (9.61–15.60)*** | **11.29 (8.81–14.46)*** |
| Accidents | 48 (0.2) | 2.6 | 90 (0.1) | 1.2 | **2.17 (1.53–3.08)*** | **1.69 (1.18–2.43)*** |
| Natural causes | 555 (2.5) | 30.0 | 1909 (2.2) | 25.4 | **1.19 (1.08–1.30)*** | 0.93 (0.84**–**1.02) |
| Male | (n = 6990) |  | (n = 27,960) |  |  |  |
| All-cause | 496 (7.1) | 88.0 | 1371 (4.9) | 59.8 | **1.48 (1.33–1.64)*** | **1.12 (1.01–1.25)*** |
| Unnatural causes | 143 (2.0) | 25.4 | 123 (0.4) | 5.4 | **4.73 (3.72–6.02)*** | **4.22 (3.29–5.41)*** |
| Suicides | 83 (1.2) | 14.7 | 44 (0.2) | 1.9 | **7.67 (5.32–11.05)*** | **7.07 (4.85–10.28)*** |
| Accidents | 42 (0.6) | 7.5 | 65 (0.2) | 2.8 | **2.63 (1.79–3.88)*** | **2.32 (1.55–3.46)*** |
| Natural causes | 353 (5.1) | 62.7 | 1248 (4.5) | 54.5 | **1.16 (1.03–1.30)*** | **0.85 (0.75–0.96)*** |

Abbreviation: PTSD, post-traumatic stress disorder.

^1^ Event was expressed as N (percentage) and mortality rate was expressed as event per 10,000 person-years.

^2^ Model 2 was adjusted for all variables (birth year, sex, income level, urbanization level, and Charlson Comorbidity Index).

**eTable 3.** The risk of all-cause and cause-specific mortality between post-traumatic stress disorder group and unexposed group, by age

| Characteristics | PTSD, event | PTSD, mortality rate | Unexposed group, event | Unexposed group, mortality rate | Crude hazard ratio (model 1) | Adjusted hazard ratio (model 2) |
| --- | --- | --- | --- | --- | --- | --- |
| Children/adolescents (6–18) | (n = 2909) |  | (n = 11,616) |  |  |  |
| All-cause | 37 (1.3) | 14.7 | 37 (0.3) | 3.7 | **4.01 (2.54****–6.32)*** | **3.66 (2.29–5.86)*** |
| Unnatural causes | 24 (0.8) | 9.6 | 20 (0.2) | 2.0 | **4.81 (2.66–8.71)*** | **4.61 (2.50–8.50)*** |
| Suicides | 16 (0.6) | 6.4 | 8 (0.1) | 0.8 | **8.02 (3.43–18.74)*** | **8.75 (3.65–20.93)*** |
| Accidents | 5 (0.2) | 2.0 | 11 (0.1) | 1.1 | 1.83 (0.63–5.25) | 1.62 (0.55–4.81) |
| Natural causes | 13 (0.4) | 5.2 | 17 (0.1) | 1.7 | **3.06 (1.49–6.30)*** | **2.58 (1.22–5.46)*** |
| Adults (18–65) | (n = 24,413) |  | (n = 97,655) |  |  |  |
| All-cause | 1018 (4.2) | 49.3 | 2092 (2.1) | 24.9 | **1.99 (1.84–2.14)*** | **1.55 (1.44–1.68)*** |
| Unnatural causes | 460 (1.9) | 22.3 | 268 (0.3) | 3.2 | **6.97 (5.99–8.10)*** | **6.07 (5.20–7.09)*** |
| Suicides | 318 (1.3) | 15.4 | 115 (0.1) | 1.4 | **11.22 (9.06–13.89)*** | **10.34 (8.32–12.86)*** |
| Accidents | 76 (0.3) | 3.7 | 116 (0.1) | 1.4 | **2.66 (2.00–3.56)*** | **2.17 (1.61–2.93)*** |
| Natural causes | 558 (2.3) | 27.0 | 1824 (1.9) | 21.7 | **1.25 (1.14–1.37)*** | 0.94 (0.85–1.03) |
| Older adults (≥65) | (n = 1455) |  | (n = 5837) |  |  |  |
| All-cause | 359 (24.7) | 369.1 | 1356 (23.2) | 338.2 | **1.13 (1.00–1.27)*** | **0.85 (0.75–0.95)*** |
| Unnatural causes | 22 (1.5) | 22.6 | 40 (0.7) | 10.0 | **2.30 (1.37–3.88)*** | **1.72 (1.01–2.95)*** |
| Suicides | 12 (0.8) | 12.3 | 8 (0.1) | 2.0 | **6.18 (2.52–15.13)*** | **4.42 (1.77–11.07)*** |
| Accidents | 9 (0.6) | 9.3 | 28 (0.5) | 7.0 | 1.36 (0.64–2.89) | 1.02 (0.47**–**2.20) |
| Natural causes | 337 (23.2) | 346.5 | 1316 (22.5) | 328.2 | 1.09 (0.97–1.23) | **0.82 (0.73–0.93)*** |

Abbreviation: PTSD, post-traumatic stress disorder.

^1^ Event was expressed as N (percentage) and mortality rate was expressed as event per 10,000 person-years.

^2^ Model 2 was adjusted for all variables (birth year, sex, income level, urbanization level, and Charlson Comorbidity Index).

**eFigure 1.** Cumulative incidence curves in post-traumatic stress disorder group and unexposed group

**A) All-cause
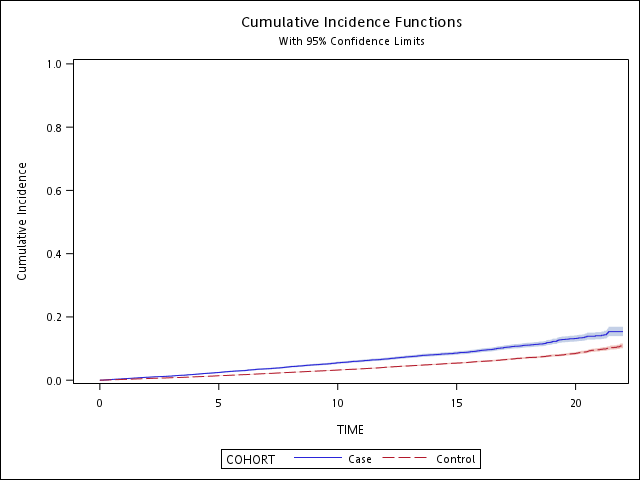
**

**B) Unnatural causes**

**
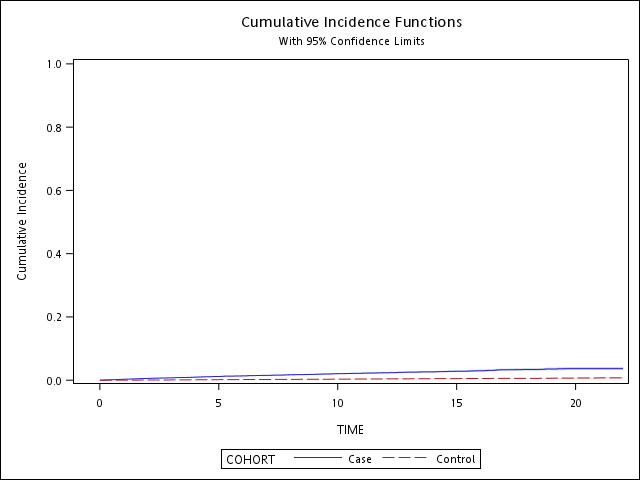
**

**C) Suicides**

**
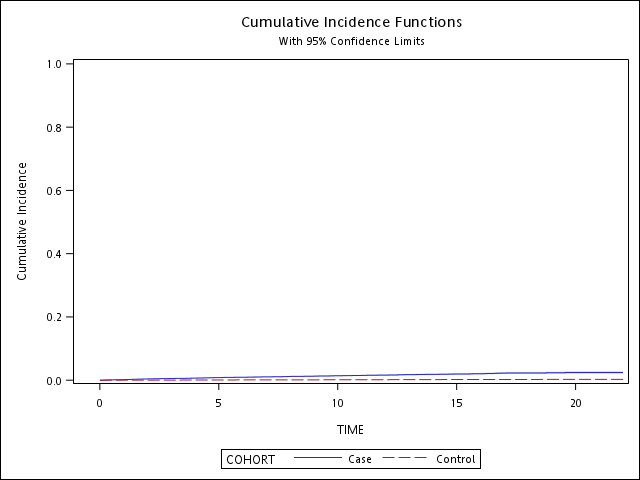
**

**D) Accidents
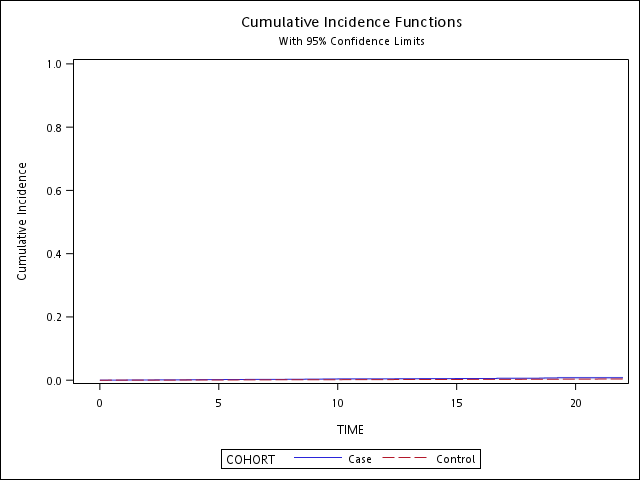
**

**E) Natural causes**

**
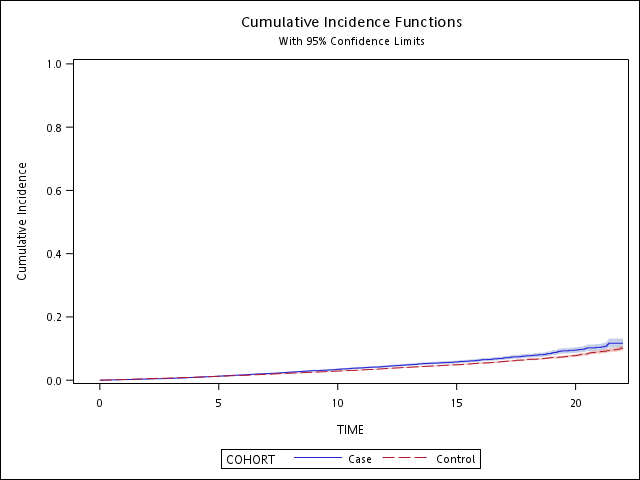
**
